# Precise Measurement and Compensation of the Micro Product of Inertia for Float Assembly in Pendulous Integrating Gyroscopic Accelerometers

**DOI:** 10.3390/s23031564

**Published:** 2023-02-01

**Authors:** Xiaojun Zhou, Gongliu Yang, Wentao Niu, Yongqiang Tu

**Affiliations:** 1 School of Instrumentation and Optoelectronic Engineering, Beihang University, Beijing 100191, China; 2 Beijing Institute of Aerospace Control Devices, Beijing 100039, China

**Keywords:** pendulous integrating gyroscopic accelerometer (PIGA), micro product of inertia (MPOI), quadratic error, float assembly, measurement and compensation

## Abstract

Nonlinear error has become the most critical factor restricting the measurement accuracy of pendulous integrating gyroscopic accelerometers (PIGA) during their improvement. The key to nonlinear error suppression for PIGA is the precise measurement and compensation of the micro product of inertia (MPOI) of the float assembly. However, the existing equipment and procedure for product of inertia (POI) measurement and compensation do not meet the accuracy requirements for MPOI. To solve this problem, novel equipment and procedures are proposed for the measurement and compensation of MPOI. The principle of the proposed measurement method is to simulate the error produced by MPOI in PIGA by using a single-axis turntable to rotate the float assembly along the eccentric axis to generate a centrifugal moment due to MPOI. The principle of the proposed compensation method is to remove the asymmetric mass to reduce the MPOI to zero. Through experimental validation, it is concluded that: (1) the measurement and compensation accuracy of the proposed method are better than 1 × 10^−10^ kg·m^2^ and 3 × 10^−10^ kg·m^2^, respectively; (2) the proposed method is validated as the MPOI is reduced from 7.3 × 10^−9^ kg·m^2^ to 3 × 10^−10^ kg·m^2^ for a real float assembly in PIGA, and the quadratic error of PIGA is reduced from 10^−5^/g_0_ to 3 × 10^−7^/g_0_.

## 1. Introduction

Pendulous integrating gyroscopic accelerometers (PIGA), which provide acceleration information named as a specific force for vehicles [1,2], is one type of core inertial sensor used in inertial navigation systems (INS). Although other types of accelerometer for INS, including quartz flexible accelerometers (QFA) [3,4], microelectromechanical systems (MEMS) capacitive accelerometers [5,6] and resonant accelerometers [7,8] have the advantages of simple structures and low cost compared with PIGA, PIGA is still an irreplaceable inertial sensor for supporting INS in applications with ultra-high navigation and guidance accuracy requirements such as intercontinental ballistic missiles (ICBM) and ocean-going submarines [9]. This is owing to its outstanding advantages of much higher precision, good linearity, strong stability and resistance to electromagnetic shock [10]. The navigation accuracy of INS is restricted to the measurement error of PIGA as the velocity and position are obtained by one integration and quadratic integration of the output of PIGA, respectively [11,12,13].

The measurement error of PIGA is mainly caused by bias, scale factor and nonlinear error [14,15]. In the past few decades, the error coefficients of the bias and scale factor of PIGA have been fully studied and effectively reduced [16,17,18]; and satisfactory results have been achieved as the bias stability of PIGA can now be reduced to 0.1 µg and scale factor stability is better than 0.1 ppm [19,20]. Thus, nonlinear error has become the most critical factor restricting the measurement accuracy of PIGA with its improvement [21]. In recent years, many scholars have carried out research into nonlinear error calibration for PIGA by high-acceleration calibration methods using precision centrifuges, vibrators and rocket sleds, which contribute greatly to providing accurate methods for the calibration of the nonlinear error of PIGA at instrument level [22,23,24,25].

However, previous works on the nonlinear error calibration of PIGA lack studies of error compensation after calibration. Also, the suppression of nonlinear error of PIGA at the system level is a challenge as the error transmission mechanisms at system level are very complicated when PIGA is installed in INS [26]. Thus, to improve the navigation accuracy of PIGA-based INS, it is critical to suppress the nonlinear error of PIGA at the instrument level. According to an analysis of nonlinear error of PIGA in the literature [27], the nonlinear error includes a quadratic term error caused by unequal inertia and product of inertial (POI) of the float assembly, a cross-coupling error caused by lateral accelerations and an error caused by unequal stiffness; and the POI of the float assembly is the most critical factor contributing to nonlinear error. Thus, the key to nonlinear error suppression for PIGA at instrument level is the precise measurement and compensation of the POI of the float assembly so that the POI is close to zero.

The measurement of POI belongs in the category of measurement of inertial parameters (i.e., mass, center of gravity (COG) and inertia tensor including moment of inertia (MOI) and POI) [28]. Various types of equipment have been developed for measuring POI using different identification methods including static and dynamic methods [29]. For example, one paper [30] described the equipment and procedure for determination of a large-object inertia parameter based on a rigid-body complete motion equation and least-squares optimization. Barreto et al. [31] proposed a particular three-dimensional (3D) trajectory that improves the experimental measurement of the inertia tensor of rigid bodies. Fakhari et al. [32] investigated disturbances in a POI measurement system theoretically and experimentally and then proposed solutions to eliminate disturbances and improve accuracy for the POI measurement system. Another paper [33] described the development and operation of a novel instrumented torsion platform used to estimate the inertia tensor of small objects with complex geometries. All these equipment and methods are suitable for measurement of the POI of large complex mechanical systems such as ground vehicles, satellites, airplanes or ships [34]. Usually, many factors that affect the POI measurement accuracy such as the leveling error of the measuring platform, the processing and assembly error of the tooling, and the air damping generated when the product is twisted are ignored in the existing equipment for POI measurement. As the measurement object generally has a relatively simple structure, even if the above-mentioned factors are ignored, the POI measurement accuracy can reach the required value. Also, the measurement accuracy of the existing POI measurement equipment is usually in the order of 10^−4^ kg·m^2^.

However, for the float assembly in PIGA, the structure is precise and complex, and the POI is the micro product of inertia (MPOI), which is in the order of 10^−9^ kg·m^2^. Any small input error will cause a large deviation in the MPOI measurement results for the float assembly. It is difficult to meet the accuracy requirement of the MPOI measurement for float assembly in PIGA with the existing equipment and procedures for POI measurement. Therefore, to reduce quadratic error and improve measurement precision of PIGA at the instrument level, it is critical to propose a precise measurement and compensation method for the MPOI of the float assembly in PIGA.

The remainder of the paper is organized as follows. Section 2 studies the impact of MPOI in PIGA. Section 3 proposes the equipment and procedure for MPOI measurement. Section 4 proposes the equipment and procedure for MPOI compensation. The experimental validations are in Section 5. The conclusions are given in Section 6.

## 2. Impact of MPOI on PIGA

### 2.1. Error Analysis

**Figure 1:**
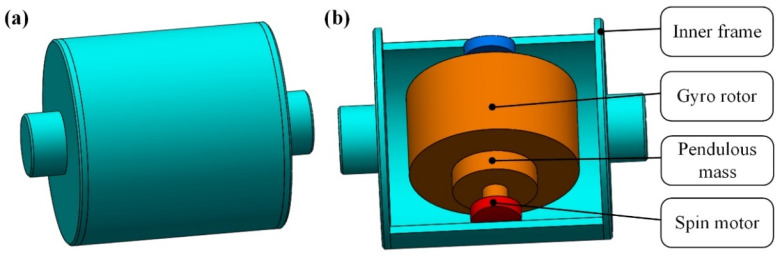
Structure of the float assembly in PIGA: (**a**) appearance of the float assembly; (**b**) composition of the float assembly.

As shown in Figure 1, the float assembly in PIGA consists of an inner frame which is of cylindrical shape, a gyro rotor with a fixed pendulous mass, and a spin motor.

**Figure 2:**
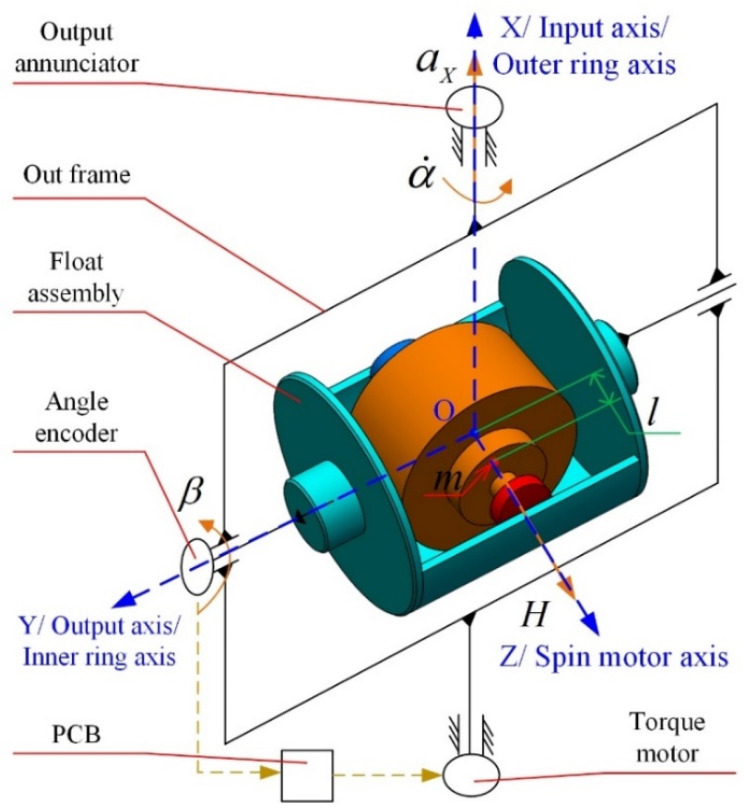
Working principle and established coordinate of PIGA.

As illustrated in Figure 2, the working principle of PIGA is as follows:

The gyro rotor is driven to rotate at a constant angular speed by the spin motor, and the angular momentum of the gyro rotor is H. The pendulous mass fixed in the gyro rotor is denoted by m, and l is the displacement between the COG of the gyro rotor and the pendulous mass. Acceleration $a_{X}$ along the input axis, which is also the outer ring axis, causes a corresponding torque $mla_{X}$ on the gyro rotor about the output axis, which is also the inner ring axis, to cause a precession motion of the outer frame about the outer ring axis. The output annunciator is used to measure the precession angular rate $\dot{\alpha }$ of the out-frame. An angle encoder is used to measure β, which is the rotation angle of the float assembly about the inner ring axis due to the friction torque and other disturbance torques. The PCB is used to control the torque motor based on the value of β to offset the interference torque. Finally, $\dot{\beta }$, $\ddot{\beta }$ ≈ 0, and the acceleration $a_{X}$ can be obtained using the equation as follows:

(1) $$H\dot{\alpha }=mla_{X}$$

PIGA is a mechanical inertial instrument, and its accuracy is determined by the stability of its mechanical structure, as the speed of the out-frame’s precession indicates the magnitude of the sensitive acceleration directly. The order of magnitude of the quadratic term coefficient due to structural instability is 10^−6^/g_0_. Meanwhile, the order of magnitude of the quadratic term coefficient due to digital measurement error is 10^−9^/g_0_. Thus, digital measurement errors of PIGA can be neglected in comparison with the measurement error due to structural instability.

As shown in Figure 2, coordinate O−XYZ is established for error analysis of PIGA. Point O is the center of mass of the gyro rotor, which is also the intersection of the inner ring axis and outer ring axis. Axis OX coincides with the outer ring axis; axis OY coincides with the inner ring axis; and axis OZ coincides with the spin motor axis. For PIGA installed on the platform INS (PINS), the error caused by the implicated motion due to angular motion of the base can be ignored. Therefore, according to the kinematics and dynamics analyses of PIGA in the coordinate O−XYZ, the expression of $\dot{\alpha }$ can be obtained as shown in Equation (2).

(2) $$\dot{\alpha }=\frac{M_{YR}}{H}+\frac{ml}{H}a_{X}+\frac{ml}{H}(a_{Y}\sin\alpha -a_{Z}\cos\alpha )\beta +\frac{1}{H}\left[(I_{X}-I_{Z})\beta +J_{XZ}\right]{\left(\frac{ml}{H}\right)}^{2}a_{X}{}^{2}$$

where $\dot{\alpha }$ is the precession angular rate of the out-frame; $M_{YR}$ is the inner frame disturbance torque; H is the angular momentum of the gyro rotor; m is the pendulous mass fixed in the gyro rotor; l is the displacement between the center of mass of the gyro rotor and the pendulous mass; $a_{X}$, $a_{Y}$ and $a_{Z}$ are projections of input acceleration on axes OX, OY and OZ of the coordinate O−XYZ, respectively; α is the precession angular of the out-frame; β is the rotation angle of the float assembly about the inner ring axis; $I_{X}$ and $I_{Z}$ are the MOI of the float assembly about axes OX and OZ, respectively; $J_{XZ}$ is the MPOI of the float assembly on the plane XOZ.

Further, as shown in Equation (3), the differential equation of float assembly motion in PIGA is obtained based on Equation (2).

(3) $$\dot{\alpha }=K_{0}+K_{1}a_{X}+K_{2}a_{X}^{2}+\frac{ml}{H}\beta (\sin\alpha \cdot a_{Y}-\cos\alpha \cdot a_{Z})$$

where $K_{0}$=$\frac{M_{YR}}{H}$ is the bias; $K_{1}$=$\frac{ml}{H}$ is the scale factor; $K_{2}$ is the quadratic error coefficient as shown in Equation (4).

(4) $$K_{2}=\frac{1}{H}\left[(I_{X}-I_{Z})\beta +J_{XZ}\right]{\left(\frac{ml}{H}\right)}^{2}$$

The error of $K_{0}$ and $K_{1}$ has been well studied and suppressed for PIGA [35], and $K_{2}$ is the main error coefficient for the current PIGA. According to Equation (4), $K_{2}$ is caused by two factors: (1) the unequal inertia $(I_{X}-I_{Z})\beta$; and (2) MPOI $J_{XZ}$. The unequal inertia $(I_{X}-I_{Z})\beta$ can be effectively suppressed by reducing β to 0. However, MPOI $J_{XZ}$ should be measured first and then compensated based on the measurement result to suppress the impact of $J_{XZ}$ on PIGA.

For example, for a certain PIGA, β is controlled as 1 arc second and the value of $\left(I_{X}-I_{Z}\right)$ is 4 × 10^−7^ kg·m^2^, and the value of $J_{XZ}$ is 5 × 10^−9^ kg·m^2^. Using Equation (4), the quadratic error coefficients caused by $(I_{X}-I_{Z})\beta$ and $J_{XZ}$ are 8 × 10^−8^/g_0_ and 5 × 10^−6^/g_0_, respectively. Thus, MPOI $J_{XZ}$ is the most critical factor for the quadratic error coefficient, and it is necessary to measure and compensate the MPOI of the float assembly to improve the accuracy of PIGA.

### 2.2. Source of MPOI

The MPOI $J_{XZ}$ of the float assembly is caused by mass asymmetry on the plane XOZ. To minimize the MPOI, the float assembly should be designed to be symmetrical to the XOZ plane. However, due to structural limitations and processing and assembly error, it is difficult to ensure that the MPOI is completely zero in engineering. The main sources of MPOI are as follows:

(1) Asymmetry in structural design: for example, the inflation nozzle can be designed at only one end of the float assembly, and the MPOI caused by this asymmetry is 4.2 × 10^−10^ kg·m^2^;
(2) Inhomogeneity of material: if there is inhomogeneity in the material in the OX or OZ axis directions, it will produce an MPOI in the order of 10^−9^ kg·m^2^;
(3) Processing error of parts: processing errors such as asymmetry, roundness and concentricity of parts will produce an MPOI; for example, if the asymmetry of the inner frame along the OZ axis is 0.01 mm, the MPOI will be 2 × 10^−10^ kg·m^2^;
(4) Assembly error: assembly errors such as fit clearance, installation error and asymmetry of glue coating, for example, the positioning gap deviation in the OZ axis direction, will cause an MPOI which is in the order of 10^−9^ kg·m^2^.

The MPOI produced by the above various asymmetric masses on the float assembly could reach a maximum value of 8 × 10^−9^ kg·m^2^. However, the accuracy of existing equipment and procedures of POI measurement is in the order of 10^−4^ kg·m^2^, which does not meet the requirements for MPOI measurement. Therefore, in this paper, novel equipment and procedure for MPOI measurement are proposed, and compensation is conducted based on MPOI measurement.

The scientific novelty of the paper is in proposing the novel measurement and compensation equipment and procedures for the MPOI of a float assembly in PIGA, which is in the order of 10^−9^ kg·m^2^. Based on this scientific novelty, the MPOI of a float assembly in PIGA can be precisely measured and compensated, which will greatly improve the measurement accuracy of PIGA.

## 3. Measurement of MPOI

### 3.1. Measurement Principle

The MPOI of the float assembly is very small, in the order of 10^−9^ kg·m^2^, and it is very difficult to measure it directly and accurately. Thus, the MPOI should be excited and amplified to achieve the required resolution and measurement accuracy. Meanwhile, the interference of other POI should be avoided to ensure that the float assembly is in a stable state in the measurement.

This paper proposes a precise measurement method for the MPOI of a float assembly in PIGA by simulating the error produced by MPOI using a single-axis turntable to rotate the float assembly along the eccentric axis to generate a centrifugal moment due to MPOI. This method maximizes the tiny centrifugal moment generated by the MPOI of the float assembly, which can effectively avoid the interference of the yaw moment on the float assembly in the gravity field environment.

**Figure 3:**
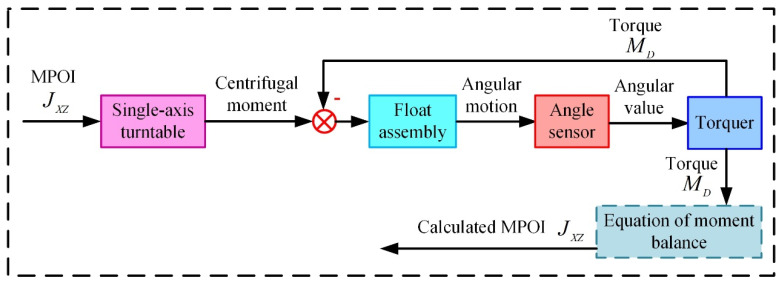
Proposed measurement principle for MPOI of float assembly.

**Figure 4:**
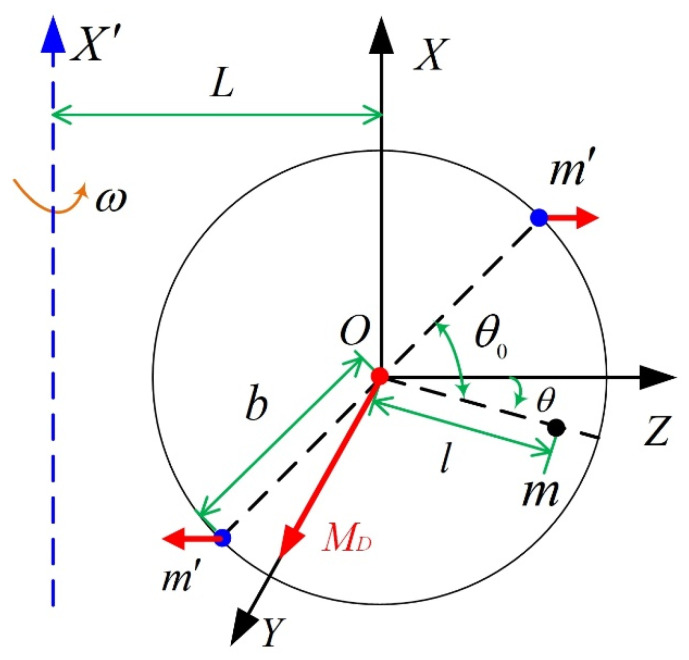
Illustration of rotation of float assembly in single-axis turntable.

The measurement principle is plotted in Figure 3, and the rotation of the float assembly in a single-axis turntable is illustrated in Figure 4. As shown in Figure 3 and Figure 4, the float assembly has the MPOI $J_{XZ}$, and the parallel distance between the $X^{\prime }$ axis (the rotation axis of the turntable) and the OX axis is L. The single-axis turntable drives the float assembly to rotate at a constant angular velocity ω around the $X^{\prime }$ axis to generate a centrifugal moment around the inner ring axis caused by the MPOI. The rotation angle around the inner ring axis of the float assembly due to the generated centrifugal moment is measured by the angle sensor, and a torquer applies torque $M_{D}$ to the float assembly by feedback control to balance the centrifugal moment due to the MPOI. Finally, the MPOI $J_{XZ}$ is calculated using the equation of moment balance.

As illustrated in Figure 4, m′ and b are the equivalent mass and the equivalent eccentricity that produce the MPOI, respectively; θ is the rotation angle around the inner ring axis for the float assembly; $\theta_{0}$ is the angle between m and m′. Thus, the MPOI $J_{XZ}$ can be defined using Equation (5).

(5) $$J_{XZ}=2m\prime b^{2}\sin\theta_{0}\cos\theta_{0}$$

Meanwhile, the equation of moment balance can be written as follows:

(6) $$\begin{array}{l} m\prime \left[L+b\cos\left(\theta_{0}-\theta \right)\right]\cdot \omega^{2}\cdot b\sin\left(\theta_{0}-\theta \right)-m\prime \left[L-b\cos\left(\theta_{0}-\theta \right)\right]\cdot \omega^{2}\cdot b\sin\left(\theta_{0}-\theta \right)\mp \\ m\left(L\pm l\cos\theta \right)\omega^{2}l\sin\theta =M_{D} \end{array}$$

where ∓ and ± are − and +, respectively, when the $X^{\prime }$ axis and m are on both sides of the OX axis; ∓ and ± are + and −, respectively, when $X^{\prime }$ axis and m are on one side of the OX axis.

As centrifugal moment is balanced by torque $M_{D}$, θ≈0 and Equation (7) can be obtained by combining Equations (5) and (6).

(7) $$J_{XZ}=\frac{M_{D}}{\omega^{2}}$$

Thus, the MPOI $J_{XZ}$ can be measured using the feedback torque $M_{D}$ of the torquer and Equation (7).

### 3.2. Equipment of MPOI Measurement

**Figure 5:**
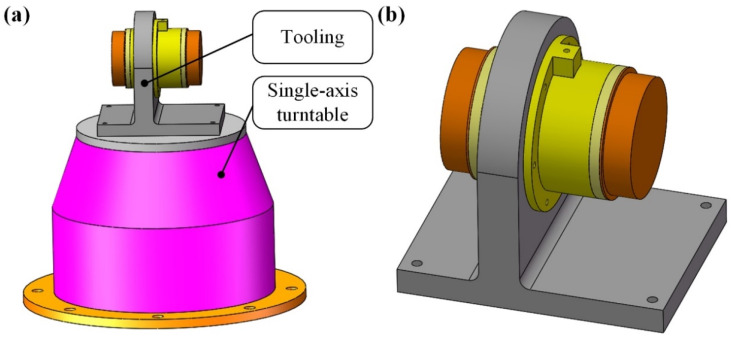
Designed equipment of MPOI measurement for float assembly in PIGA: (**a**) composition of equipment; (**b**) appearance of tooling.

According to the proposed measurement principle, novel equipment for MPOI measurement of the float assembly in PIGA is designed as shown in Figure 5. The equipment consists of a tooling and a single-axis turntable.

**Figure 6:**
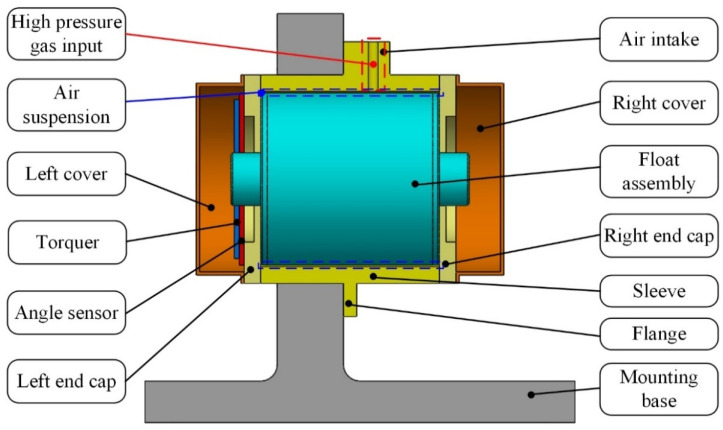
Composition and structure of the tooling.

As shown in Figure 6, the tooling consists of a mounting base, a sleeve, a left end cap, a right end cap, an angle sensor, a torquer, a left cover and a right cover. The sleeve is mounted on the mounting base by a flange. The left end cap and right end cap are mounted on the sleeve to form a closed cylindrical space; and the float assembly is mounted in a closed cylindrical space. High-pressure gas is inputted through the air intake to suspend the float assembly by air suspension between the outer surface of the float assembly and the inner surface of the sleeve. The angle sensor and the torquer are mounted on the left end cap to measure the angle value of float assembly around the inner ring axis and to apply torque to balance the centrifugal moment, respectively. The left cover and the right cover are mounted on the left end cap and right end cap, respectively, to protect the float assembly from dust and temperature shock.

### 3.3. Measurement Error Analysis

(1) Measurement principle error

When the float assembly rotates a small angle θ around the inner ring axis, the original MPOI $J_{XZ}$ will change, and the new MPOI ${J^{\prime }}_{XZ}$ can be written as Equation (8) according to the coordinate transformation.

(8) $$J_{XZ}{}^{\prime }=AJ_{XZ}A^{T}$$

where A=$\left[\begin{array}{ccc} \cos\theta & 0 & \sin\theta \\ 0 & 1 & 0\\ -\sin\theta & 0 & \cos\theta \end{array}\right]$ is the coordinate transformation matrix. Thus, the new MPOI ${J^{\prime }}_{XZ}$ and the measurement principle error η can be written as Equations (9) and (10), respectively.

(9) $$J_{XZ}^{\prime }=\cos\theta \left(J_{XZ}\cos\theta +I_{Z}\sin\theta \right)-\sin\theta \left(I_{X}\cos\theta +J_{XZ}\sin\theta \right)$$

(10) $$\eta =\frac{J_{XZ}^{\prime }-J_{XZ}}{J_{XZ}}=\cos2\theta -1+\frac{I_{Z}-I_{X}}{2J_{XZ}}\sin2\theta$$

Because the value of θ is set at less than 5 arc seconds, which is approaching zero, the measurement principle error η also approaches zero, so it can be ignored.

(2) Measurement error caused by gravity

The unsteady component of the change in direction due to the gravitational component during rotation is mglsinθ. In order to reduce the impact of this error on the measurement result, the unsteady component should be much smaller than the centrifugal moment generated by the MPOI, as follows:

(11) $$mgl\sin\theta \ll ml\omega^{2}\left(L+l\cos\theta \right)\sin\theta$$

Equation (11) can be simplified as follows:

(12) $$\omega^{2}\left(L+l\cos\theta \right)\gg g$$

It can be seen that the higher the ω and the larger the L, the smaller the measurement error due to gravity. When ω is set at 30 rad/s and L is set at 300 mm, the centrifugal moment is 27 times that of the unsteady component due to gravity, and the measurement error caused by gravity can be ignored.

(3) Measurement error caused by installation error

Assuming that the installation error angle between the $X^{\prime }$ axis and the OX axis is δ, Equation (6) is revised into Equation (13).

(13) $$\begin{array}{l} m\prime \left[L+b\cos\left(\theta_{0}-\theta -\delta \right)\right]\cdot \omega^{2}\cdot b\sin\left(\theta_{0}-\theta -\delta \right)-m\prime \left[L-b\cos\left(\theta_{0}-\theta -\delta \right)\right]\cdot \omega^{2}\cdot b\sin\left(\theta_{0}-\theta -\delta \right)\mp \\ m\left[L\pm l\cos\left(\theta +\delta \right)\right]\omega^{2}l\sin\left(\theta +\delta \right)=M_{D} \end{array}$$

As both θ and δ are small quantities, Equation (14) can be obtained by combining Equations (5) and (13).

(14) $$J_{XZ}=\frac{M_{D}}{\omega^{2}}+ml\left(L\pm l\right)\left(\theta +\delta \right)$$

Thus, the measurement error caused by installation error is ml(L±l)(θ+δ), so it can be ignored, as δ is controlled at the arc second level.

(4) Measurement error caused by the support disturbance moment

During measurement, the support disturbance moment on the float assembly will produce a measurement error of MPOI. Assuming that the measurement accuracy of MPOI is 10^−9^ kg·m^2^ and the rotation speed of the single-axis turntable is 600 r/min, the centrifugal moment generated by the MPOI of this precision level is calculated as M = 4 × 10^−7^ N·m. The support disturbance moment on the float assembly should be less than M/3 = 1.3 × 10^−7^ N·m.

In order to reduce the impact of the support disturbance moment on measurement accuracy, the equipment uses air suspension to support the float assembly as shown in Figure 6. The support disturbance moment is measured as follows: the float assembly is not installed in the tooling, and high-pressure gas is inputted through the air intake at the designed pressure value; the output of the torquer is the support disturbance moment due to eddy current interference. The measurement result of the support disturbance moment is 8.3 × 10^−8^ N·m, which meets the requirement of less than 1.3 × 10^−7^ N·m. Thus, measurement error caused by the support disturbance moment can be ignored for the proposed equipment.

In summary, measurement error caused by the measurement principle, gravity, installation error and support disturbance moment is very small, and can be ignored for the proposed measurement equipment and procedure.

## 4. Compensation of MPOI

After the MPOI is measured, compensation of MPOI is conducted based on the measurement result to reduce MPOI in order to reduce the quadratic error of PIGA.

### 4.1. Compensation Principle

**Figure 7:**
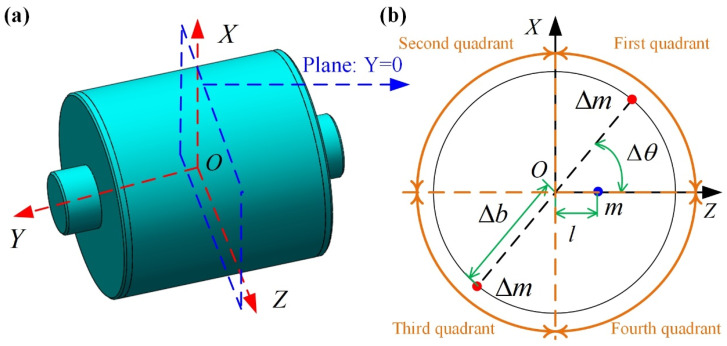
Compensation of MPOI of float assembly: (**a**) the plane of mass adjustment; (**b**) details of mass removal on the plane.

For the inherent MPOI caused by asymmetrical mass distribution, the MPOI can be compensated by removing the asymmetric mass to theoretically reduce the value of MPOI to zero. To minimize the impact of the adjusted mass on the pendulum, liquid floating balance and operating temperature of the float assembly, mass adjustment of two symmetrical points on the plane Y = 0 of the float assembly is performed as shown in Figure 7.

As shown in Figure 7b, if the measured $J_{XZ}$ is positive, the removed mass is in the first and third quadrants; if the measured $J_{XZ}$ is negative, the removed mass is in the second and fourth quadrants. The adjusted MPOI $\Delta J_{XZ}$ by mass removal is:

(15) $$\Delta J_{XZ}=2\Delta m\Delta b^{2}\sin\Delta \theta \cos\Delta \theta =\Delta m\Delta b^{2}\sin2\Delta \theta$$

where $\Delta J_{XZ}$ is the adjusted MPOI by mass removal; Δm is the removed mass; Δb is the distance between point O and the position of mass removal; Δθ is the angle between the removed mass and the pendulous mass.

To minimize Δm, Δθ is set as 45° and Δb is set as $r_{f}$ ($r_{f}$ is the radius of the float assembly on the plane Y=0). Thus, the removed mass Δm is calculated as follows:

(16) $$\Delta m={\left(J_{XZ}\right)}_{m}/r_{f}{}^{2}$$

where ${\left(J_{XZ}\right)}_{m}$ is the measurement result of the MPOI.

### 4.2. Compensation Equipment

**Figure 8:**
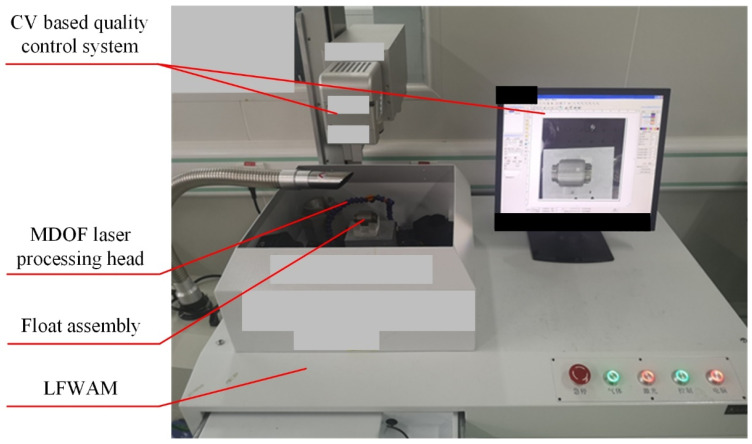
The developed LFWAM for the mass removal of float assembly in PIGA.

A laser fine-weight adjusting machine (LFWAM) is developed for the mass removal of the float assembly in PIGA as shown in Figure 8. The main components of the LFWAM are a multi-degree-of-freedom (MDOF) laser processing head and a computer-vision-based (CV-based) quality-control system. The MDOF laser processing head controlled by a computer program realizes the precise positioning and mass removal on complex surfaces with high processing and positioning accuracy based on the CV-based quality-control system. In mass-removal processing, the LFWAM uses a laser pulse with a short width and high peak power to rapidly heat up, melt and vaporize the locally removed surface of the float assembly to achieve the precise removal of surface material. As the pulse action time and laser pulse width are very short, on the ns level, the heat-affected zone formed in the removed material is very shallow, on the µm level, so the mass removal process is a “quasi-cold state” which will not affect the surface quality and stress around the float assembly. The developed LFWAM can achieve a correction accuracy of 0.01 mg mass and 5 × 10^−10^ kg·m^2^ MPOI on the surface of the float assembly, which meet the correction accuracy requirements of the MPOI of the float assembly in PIGA.

## 5. Experimental Validations

In order to validate the proposed equipment and procedure of the measurement and compensation for the MPOI of the float assembly in PIGA, experimental validations for measurement accuracy, compensation accuracy, and measurement and compensation of a real float assembly are conducted.

### 5.1. Experimental Validation for Measurement Accuracy

To validate the measurement accuracy, a standard cylinder with the same structure and shape as the float assembly is used to measure the MPOI using the proposed equipment and procedure of MPOI measurement. For the same standard cylinder, repeated disassembly and MPOI measurement tests in the tooling are carried out a total of six times. The test results are listed in Table 1.

The repeatability standard deviation of the six tests is 0.83 × 10^−10^ kg·m^2^. Thus, the measurement accuracy of the proposed equipment and procedure of the MPOI are validated as a measurement accuracy of the MPOI of better than 1 × 10^−10^ kg·m^2^.

### 5.2. Experimental Validation for Compensation Accuracy

**Figure 9:**
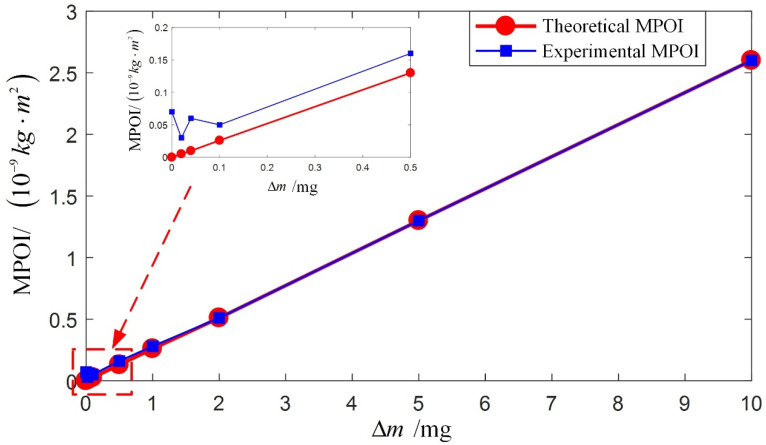
Comparison of theoretical and experimental MPOI of the standard cylinder after mass removal.

The standard cylinder is continued to be used to validate the compensation accuracy of the proposed equipment and procedure of MPOI compensation. In the experimental validation, mass removals are conducted on the standard cylinder, and the theoretical MPOI is calculated using Equation (16). Experimental MPOI of the standard cylinder after each mass removal is measured using the proposed measurement equipment and procedure. The theoretical and experimental MPOI are listed in Table 2 and plotted in Figure 9.

As shown in Figure 9, when the change in MPOI due to mass removal is less than 1 × 10^−10^ kg·m^2^, the measured value of the MPOI is randomly distributed and the displayed value is the main error. When the change in MPOI due to mass removal is greater than 1 × 10^−10^ kg·m^2^, the measured value of the MPOI will significantly change with the removed mass. When the change in MPOI due to mass removal is greater than 3 × 10^−10^ kg·m^2^, the measured value of the MPOI will strictly correspond to the removed mass. Thus, it can be concluded that the compensation accuracy of the MPOI using the proposed equipment and procedure is validated and is better than 3 × 10^−10^ kg·m^2^ and in the same order as the measurement accuracy.

### 5.3. Measurement and Compensation of Float Assembly

After the accuracy of measurement and compensation of MPOI for the standard cylinder is validated, measurement and compensation of the real float assembly in PIGA are conducted to validate the proposed equipment and procedure. Measurement of MPOI of the float assembly is conducted using the tooling and single-axis turntable as shown in Figure 5. After the MPOI is measured, it is compensated using the LFWAM as shown in Figure 8.

Six float assemblies are selected to conduct the experimental validation. Before measurement and compensation of the MPOI, the quadratic error of PIGA is approximately 10^−5^/g_0_, and the measured results of MPOI are in the range of 1.5 × 10^−9^ kg·m^2^ to 7.3 × 10^−9^ kg·m^2^. After MPOI compensation, the measured results of the compensated MPOI are below 3×10^−10^ kg·m^2^. Finally, the six float assemblies are assembled into six PIGA, and the precision centrifugal is used to calibrate the quadratic error of PIGA. The calibrated results of the quadratic error are below 3 × 10^−7^/g_0_. Thus, the proposed method of measurement and compensation of MPOI for the float assembly in PIGA is validated as the MPOI is reduced from 7.3 × 10^−9^ kg·m^2^ to 3 × 10^−10^ kg·m^2^ and the quadratic error of PIGA is reduced from 10^−5^/g_0_ to 3 × 10^−7^/g_0_. As a result, the navigation accuracy of PIGA-based PINS is improved from 3000 m to 600 m for a flight trajectory of a typical vehicle.

## 6. Conclusions

In this study, novel equipment and procedures are proposed for the measurement and compensation of MPOI for a float assembly in PIGA. Firstly, the impact of MPOI on PIGA is studied, and it is concluded that: MPOI is the most critical factor for the quadratic error coefficient, and it is necessary to measure and compensate the MPOI of the float assembly to improve the accuracy of PIGA; and the MPOI of the float assembly can reach a maximum value of 8 × 10^−9^ kg·m^2^. Then, novel equipment and procedures of measurement and compensation of MPOI are proposed, because the existing equipment and procedures of POI measurement are in the order of 10^−4^ kg·m^2^, which does not meet the requirement of MPOI measurement. The principle of the proposed measurement method is to simulate the error produced by MPOI using a single-axis turntable to rotate the float assembly along the eccentric axis to generate centrifugal moment due to MPOI. Based on the principle of MPOI measurement, the measurement equipment and procedure based on a tooling and single-axis turntable are proposed. The principle of the proposed compensation method is to remove the asymmetric mass to theoretically reduce the value of MPOI to zero. Based on the principle of MPOI compensation, compensation equipment and procedure based on an LFWAM is proposed. Through experimental validations, it is concluded that: (1) the measurement and compensation accuracy of the proposed equipment and procedure are better than 1 × 10^−10^ kg·m^2^ and 3 × 10^−10^ kg·m^2^, respectively; (2) the proposed method is validated as the MPOI is reduced from 7.3 × 10^−9^ kg·m^2^ to 3 × 10^−10^ kg·m^2^ for a real float assembly in PIGA and the quadratic error of PIGA is suppressed below 3 × 10^−7^/g_0_.

This work contributes to the precise measurement and compensation for the MPOI of a PIGA float assembly. Future work will conduct research into nonlinear error analysis and the suppression of PIGA at a system level.

## Figures and Tables

**Table 1:** Repeatability test of MPOI measurement of the standard cylinder for multiple installations.

Experiment Number	1	2	3	4	5	6
Measured MPOI (kg·m^2^)	0.7 × 10^−10^	1.3 × 10^−10^	−0.9 × 10^−10^	0.5 × 10^−10^	−0.3 × 10^−10^	−0.5 × 10^−10^

**Table 2:** Theoretical and experimental MPOI of the standard cylinder after mass removal.

Experiment Number	Removed Mass (mg)	Theoretical MPOI (kg·m^2^)	Experimental MPOI (kg·m^2^)
1	0	0	0.7 × 10^−10^
2	0.02	5.1 × 10^−12^	0.3 × 10^−10^
3	0.04	1.0 × 10^−11^	0.6 × 10^−10^
4	0.1	2.6 × 10^−11^	0.5 × 10^−10^
5	0.5	1.3 × 10^−10^	1.6 × 10^−10^
6	1	2.6 × 10^−10^	2.8 × 10^−10^
7	2	5.1 × 10^−10^	5.1 × 10^−10^
8	5	1.3 × 10^−9^	1.3 × 10^−9^
9	10	2.6 × 10^−9^	2.6 × 10^−9^

## Data Availability

Not applicable.
